# What drives attitude towards telemedicine among families of pediatric patients? A survey

**DOI:** 10.1186/s12887-016-0756-x

**Published:** 2017-01-17

**Authors:** Luisa Russo, Ilaria Campagna, Beatrice Ferretti, Eleonora Agricola, Elisabetta Pandolfi, Emanuela Carloni, Angelo D’Ambrosio, Francesco Gesualdo, Alberto E. Tozzi

**Affiliations:** Telemedicine Unit, Bambino Gesù Children’s Hospital IRCCS, Piazza S. Onofrio 4, 00165 Rome, Italy

## Abstract

**Background:**

Telemedicine has been recognized as a way to improve accessibility, quality, and efficiency of care. In view of the introduction of new telemedicine services, we conducted a survey through a self-administered questionnaire among families of children attending the Bambino Gesù Children’s Hospital IRCCS, a tertiary care children’s hospital located in Rome, Italy.

**Methods:**

We investigated sociodemographic data, clinical information, technological profile, attitude towards telemedicine, perceived advantages of telemedicine, fears regarding telemedicine, willingness to use a smartphone app providing telemedicine services and willingness to use a televisit service. Through logistic regression, we explored the effect of sociodemographic and clinical variables and technological profile on willingness of using a telemedicine app and a televisit service.

**Results:**

We enrolled a total of 751 families. Most patients had a high technological profile, 81% had at least one account on a social network. Whatsapp was the most popular messaging service (76%). Seventy-two percent of patients would use an app for telemedicine services and 65% would perform a televisit. Owning a tablet was associated with both outcome variables - respectively: OR 2.216, 95% CI 1.358–3.616 (app) and OR 2.117, 95% CI 1.415–3.168 (televisit). Kind of hospitalization, diagnosis of a chronic disease, disease severity and distance from the health care center were not associated with the outcome variables.

**Conclusion:**

Families of pediatric patients with different clinical problems are keen to embark in telemedicine programs, independently from severity of disease or chronicity, and of distance from the hospital.

**Electronic supplementary material:**

The online version of this article (doi:10.1186/s12887-016-0756-x) contains supplementary material, which is available to authorized users.

## Background

Once limited to delivering health care to patients in remote areas, telemedicine has been recognized as a way to improve accessibility, quality, and efficiency of care [1]. As a matter of fact, telemedicine has been adopted by a number of different medical specialties: cardiology [2–4], neurology [5, 6], surgery [7–9], dermatology [10–13], ophthalmology [14–17], radiology [18, 19]. In pediatrics, telemedicine has a wide range of potential applications [20].

In Italy, telemedicine services dedicated to the pediatric population are not common and are not always included in reimbursement policies, despite Internet connection being widespread among the population [21] and a variety of technical solutions being available on the market.

The Bambino Gesù Children’s Hospital IRCCS, a tertiary care academic hospital located in Rome, Italy, dedicated to pediatric patients with a wide range of health needs, is planning an enhancement of the existing telemedicine projects and an implementation of new services.

In order to innovate processes through telemedicine services, needs and expectations of the target public should be matched with the availability of technologic solutions and with feasibility of expected interventions.

The aim of our study was to identify needs and expectations of families of children regarding the use of telemedicine services and to investigate their technological and Internet profile, prior to development and implementation of specific telemedicine services.

## Methods

### Study design and population

This is a cross-sectional study. We conducted a survey, from September 2014 to January 2015, on a random sample of parents of children, aged 0–18 years, admitted to the Bambino Gesù Children’s Hospital IRCCS, Rome, Italy, both as outpatients and as inpatients, for acute or chronic diseases. The Bambino Gesù Children’s Hospital is a tertiary care academic hospital, with 607 inpatient-beds.

Participants were consecutively recruited on different weekdays by trained research nurses. Specifically, inpatients were recruited among those admitted in the Department of Pediatrics on Mondays and Tuesdays; patients admitted to Day Hospitals and outpatients were recruited on Wednesdays and Thursdays. Eligibility criteria included having a child aged 0–18 years, signing an informed consent and speaking Italian.

The study was approved by the Bambino Gesù Children’s Hospital’s ethical committee.

### Questionnaire

After signing in an informed consent, participants were invited to fill in a self-administered questionnaire evaluating the attitude towards telemedicine applications (see Additional file 1). Specifically, the questionnaire collected information about:

- socio-demographic data (age, sex, nationality, region of residence, postal code, education level, employment);
- child’s data: sex, age, number of the child’s hospital admissions during last year, number of visits by family pediatrician during last year;
- technological profile: availability of technological devices among smartphone, personal computer (PC), tablet, smartTV (i.e. a television set with integrated Internet and Web 2.0 features); availability of a blog or of a social media account among Facebook, Twitter, LinkedIn, Instagram, Google+; use of messaging software among Whatsapp, Skype, Facebook Messager; use of video calls; use of smartphone apps; use of smartphone apps for health; search of health information on the Internet;
- telemedicine attitude, measured on a 5-point Visual Analog Scale (VAS): perceived usefulness of the following services: an app allowing communication with other parents of children with the same disease; a diary for recording the child’s health status; an app for scheduling medical visits; telemonitoring; a service for transmitting telemonitoring data to a doctor; a service for consulting a doctor in case of emergency; reminders for medical visits; a service providing transmission of health data from the hospital; reminder for therapy; newsletter on health promotion;
- perceived advantages of telemedicine (measured on a 5-point VAS scale): time saving; cost saving; empowerment of patients; empowerment of families;
- fears regarding telemedicine (measured on a 5-point VAS scale): lack of trust regarding the use of monitoring devices without the presence of a physician; privacy; difficulty in using technological devices.

Moreover, we asked if the participant would use (Yes/No question):

- an app providing telemedicine services;
- a televisit service (defined as synchronous videoconference encounter between the provider and family/patient).

We extracted disease diagnosis and disease severity from patients’ electronic health record. A disease was defined as “chronic” according to the definition adopted by the Medical Subject Heading (MesH) database [22]. Disease severity was categorized on the basis of the American Society of Anesthesiologists (ASA) classification: I) normal healthy patient; II) patient with mild systemic disease; III) patient with severe systemic disease; IV) patient with severe systemic disease that is a constant threat to life.

### Analysis

We described sociodemographic variables, and prevalence for each item as mean and standard deviation (SD), median and range or proportions and 95% confidence intervals (CI), as appropriate. Proportions were calculated excluding missing values.

In the descriptive analysis, the participants’ technological profile was classified as follows:

- availability of technological equipment: low (0–1 device); medium (2 devices); high (3–4 devices);
- availability of blog/social network accounts: low (no account); medium (1–2 accounts); high (3–6 accounts);
- use of messaging services: low (0–1 service); medium (2 services); high (3 services).

At the univariate analysis, we studied through logistic regression the association of sociodemographic variables and variables regarding telemedicine-attitude with two different outcomes: 1) interest in using a televisit service, 2) interest in using a telemedicine app. Here follows a list of the exposure variables which were studied at the univariate analysis: respondent’s age (continuous), sex, education level (university degree or lower), employment (employed/not employed), living in the Rome province, hospital admission in the past year, visit by the family pediatrician in the past year, ASA category (1–2 vs 3–4), being a foreigner, child’s age, child’s sex, availability of technological devices (yes/no for each item), blog or social network account (yes/no for each item) and use of messaging services (yes/no for each item), use of videocalls (yes/no), having ever used an app, having ever used an app for health.

The multivariable analysis was performed through logistic regression, including the following as independent variables: type of hospital admission (inpatients, outpatients, or day hospital), having a chronic disease, ASA category and living within the Rome province, plus variables that resulted associated with the outcome with a *p* < 0.2. The p threshold <0.2 is commonly used as a compromise between including in the multivariate model all variables and selecting only those associated at the *p* < 0.05 level. Using the p value <0.2 should allow to include in the final model also those variables that, at the univariate analysis, are marginally associated with the outcome because of a confounding effect.

We calculated that 600 interviews would have been sufficient to estimate proportions with a precision of +/- 4%, with a confidence level of 95%, and an expected proportion of families who would use a telemedicine service of 50%.

## Results

### Sociodemographic and clinical characteristics

A total of 751 families were enrolled: 257 outpatients, 248 Day-hospital patients and 246 inpatients.

Characteristics of families included in the study are shown in Table 1.

**Table 1:** Sociodemographic and clinical characteristics

	Number	Percent
Respondent’s sex		
Female	522	70.16
Male	222	29.84
Respondent’s age		
≤ 35 years	228	30.89
36–45 years	327	44.31
≥ 46 years	183	24.80
Foreign nationality	86	11.70
High school diploma or university degree	594	81.00
University degree	230	31.38
Employed	484	67.22
Living in the municipality of Rome	271	39.45
Living in the province of Rome	414	60.26
Living in Lazio Region	524	76.27
Child’s sex		
Female	367	49.80
Male	370	50.20
Child’s age		
< 1 years	118	15.71
1–5 years	232	30.89
6–10 years	199	26.50
11–17 years	164	21.84
≥ 18 years	38	5.06
Frequency of hospital visits during last year		
Never	131	17.90
Up to 4 times	407	55.60
5 or more times	194	26.50
At least 1 hospital visit in the last year by age		
< 1 years	62	57.41
1–5 years	196	86.96
6–10 years	171	87.24
11–17 years	141	87.04
≥ 18 years	31	81.58
Visits to the pediatrician during last year		
Never	66	9.48
Up to 4 times	383	55.03
5 or more times	247	35.49
At least 1 visit with family pediatrician during last year by age		
< 1 years	90	89.11
1–5 years	213	97.26
6–10 years	172	92.47
11–17 years	135	87.66
≥ 18 years	20	55.56
Chronic disease	561	74.70
Disease severity (ASA)		
Lev. 1	208	27.73
Lev. 2	227	30.27
Lev. 3	265	35.33
Lev. 4	50	6.67

The majority of responding parents were females (70.2%) and had a mean age of 39.9 (SD 8.1). The large majority of respondents had a high level of education (50% had a high school diploma, 31% had a university degree). In a high proportion of enrolled families (75%), the child had a chronic disease.

Nearly one third of families had accessed the hospital 5 or more times in the past 12 months and 35% of them had visited the family pediatrician in the same period.

### Technological profile

The technological profile of the respondents is reported in Table 2.

**Table 2:** Technological profile

	Number	Percent
Smartphone	501	66.71
PC	548	72.97
Tablet	378	50.33
Smart TV	114	15.20
Technological equipment		
Low (0–1 device)	236	31.47
Medium (2 devices)	235	31.33
High (3–4 devices)	279	37.20
Blog	89	13.78
Facebook account	553	75.03
Twitter account	131	17.77
LinkedIn account	112	15.20
Instagram account	83	11.28
Google + account	199	27.00
Social network profile		
Low (no account)	122	19.09
Medium (1–2 accounts)	368	57.59
High (3–6 accounts)	149	23.32
WhatsApp	564	75.60
Skype	251	33.69
Facebook	442	59.25
Use of instant messaging services		
Low (0–1 messaging service)	309	41.48
Medium (2 messaging services)	263	35.30
High (3 messaging services)	173	23.22
Video calls	359	49.93
Ever used a smartphone app	530	71.91
Ever used a smartphone app for health	172	24.47

All respondents had at least one device allowing connection to the Internet. A high proportion of respondents had a computer (73%), a smartphone (67%) or a tablet (50%). Fifteen percent had a SmartTV. Moreover, more than one third of respondents had more than 3 or 4 devices connected to the web.

A high proportion of respondents (81%) had at least one account on a social network or a blog. As expected, the most popular social network was Facebook (75%), followed by Google + (27%), Twitter (17%) and LinkedIn (15%). Notably, 14% of respondents had a blog. Regarding the use of messaging services, Whatsapp was the most popular service used (76%), followed by Facebook Messenger (59%) and Skype (34%).

Half respondents used videocall services. A large proportion (72%) had used a smartphone app and one quarter had used a smartphone app with health-related contents.

### Telemedicine attitude

A total of 476 (65%) would do a televisit for the problem that caused the hospital access, while 85 (12%) would not; 515 (72%) would use an app, while only 23 (3%) would not. In both cases, one quarter of patients responded “I do not know”: 173 (24%) regarding the televisit, 181 (25%) regarding the app.

Table 3 shows perceived usefulness of specific telemedicine functions and perceived advantages of telemedicine services (the 5 point Lickert scale is presented in a synthetic format in order to overcome excessive granularity of data).

**Table 3:** Attitude towards telemedicine and perceived advantages

	None/A bit		Sufficient		Moderate/Much	
	N	%	N	%	N	%
Attitude towards telemedicine - perceived usefulness of:						
An app allowing communication with other parents of children	124	18	167	25	389	57
A diary for recording the child’s health status	67	9	122	17	541	74
An app for scheduling medical visits	15	2	62	8	663	90
A service for televisits	147	20	137	19	452	61
A telemonitoring service	98	13	117	16	513	70
A service for transmitting telemonitoring data to the doctor	56	8	95	13	589	80
A service for consulting a doctor in case of emergency	22	297	52	7	669	90
Reminders for medical visits	28	4	77	10	637	86
A service providing transmission of health data from the hospital	16	2	38	5	689	93
Reminders for therapy	110	15	107	15	519	71
A newsletter on health promotion	38	5	64	9	639	86
Perceived advantages of telemedicine						
Time saving	21	3	68	9	645	88
Cost saving	29	4	81	11	622	85
Empowerment of patients	60	8	121	17	545	75
Empowerment of families	54	7	125	17	551	75

Most respondents considered most of the functions of telemedicine, as investigated by our questionnaire, as moderately important or very important. Among the most appreciated potential functions of telemedicine were those concerning communication with the hospital: transmission of health data from the hospital (moderately/very important according to 93%), consultation of a doctor in case of emergency (moderately/very important according to 90%), scheduling of medical visits (moderately/very important according to 90%).

Concerning the advantages of telemedicine, most respondents thought that time saving was moderately/very important (88%), followed by cost saving (85%).

Finally, we asked those that had responded “No” or “I do not know” to both outcome questions (interest in televisits or health app) on their fears regarding telemedicine. Lack of trust towards telemedicine tools was the most addressed concern (30% considered it as moderately/very important), followed by fear of excessive responsibilities for the family (28%). Surprisingly, fear for privacy was not a strong concern (57% considered it of little importance or not important at all).

### Multivariable analysis

The multivariable analysis (see Tables 4 and 5) showed that willingness of performing a televisit was associated with the following outcomes: owning a PC (OR 1.61, 95% CI 1.02–2.542), owning a tablet (OR 2.117, 95% CI 1.415–3.168), having a LinkedIn account (OR 2.329, 95% CI 1.151–4.714), having the habit of performing videocalls (OR 1.865, 95% CI 1.138–3.057). On the other hand, the availability for the use of an app dedicated to telemedicine was associated with owning a PC (OR 2.835, 95% CI 1.68–4.784), owning a tablet (OR 2.216, 95% CI 1.358–3.616) and having already used an app for health (OR 2.689, 95% CI 1.323–5.465). Owning a Twitter account was negatively associated with willing to use an app (OR 0.448, 95% CI 0.208–0.963). Notably, distance from the hospital was not associated either with willingness to perform a televisit or to use an app.

**Table 4:** Multivariable analysis – dependent variable: willingness to use an app for health issued by the hospital

	OR	95% CI	p
Male	1.64	0.926–2.889	0.09
Graduated	1.28	0.737–2.225	0.38
Employed	0.68	0.383–1.189	0.17
Living in the Rome province	1.44	0.902–2.288	0.13
Foreign nationality	0.95	0.43–2.085	0.89
Smartphone	1.82	0.238–13.894	0.57
PC	2.84	1.68–4.784	<0.001
Tablet	2.22	1.358–3.616	<0.001
Smart TV	0.96	0.455–2.015	0.91
Blog	0.86	0.422–1.75	0.68
Facebook account	1.31	0.628–2.744	0.47
Twitter account	0.45	0.208–0.963	0.04
LinkedIn account	1.99	0.841–4.684	0.12
Instagram account	1.3	0.522–3.234	0.57
Google + account	1.81	0.963–3.402	0.07
Whatsapp	0.94	0.499–1.768	0.85
Skype	0.84	0.431–1.655	0.62
Facebook	0.84	0.429–1.656	0.62
Video calls	1.77	0.981–3.204	0.06
Ever used an app	1.69	0.928–3.086	0.09
Ever used an app for health	2.69	1.323–5.465	0.01

**Table 5:** Multivariable analysis – dependent variable: willingness to use a televisit service

	OR	95% CI	p
Graduated	1.03	0.652–1.633	0.89
Employed	1.06	0.685–1.623	0.81
Living in the Rome province	1.2	0.814–1.77	0.36
Foreign nationality	0.66	0.34–1.292	0.23
PC	1.61	1.02–2.542	0.04
Tablet	2.12	1.415–3.168	<0.001
Smart TV	0.97	0.54–1.752	0.93
Facebook account	1.5	0.817–2.756	0.19
Twitter account	0.75	0.399–1.403	0.37
LinkedIn account	2.33	1.151–4.714	0.02
Instagram account	0.85	0.43–1.671	0.63
Google + account	1.22	0.745–1.999	0.43
Whatsapp	0.98	0.575–1.665	0.94
Skype	0.96	0.556–1.654	0.88
Facebook	0.88	0.511–1.519	0.65
Videocalls	1.87	1.138–3.057	0.01
Ever used an app	1.29	0.768–2.151	0.34
Ever used an app for health	1.55	0.912–2.628	0.11

## Discussion

The decision leading to the creation and implementation of an innovation should be moved by the combination of three cornerstones: feasibility, viability, and desirability [23]. The development of a telemedicine project would benefit from the application of such principles. With the present study, we explored the last item: what the public - in our case, families of children attending a tertiary care children’s hospital - desires and expects.

Our results show a notable interest towards telemedicine: a large proportion of families were keen on the idea of using an app issued by the hospital and more than a half were willing to do a televisit. Willingness to use telemedicine services was transversal among participating families, and was not influenced by kind of hospitalization, presence of a chronic disease, disease severity and distance from the health care center. Such characteristic suggests that the creation of a telemedicine service can be oriented towards all kinds of patients, with no need of focusing on specific subgroups.

Attitude to the use of technologies for health management has previously been explored in adult patients. Most published studies have focused on specific patient populations and investigated heterogeneous items, with variable results. Interest towards telemedicine has been detected among cancer patients [24] and patients affected with chronic lung diseases [25], although, in some cases, the attitude towards the use of new technologies was low. In a large survey, patients affected with depression and those with a high risk of cerebrovascular disease reported a moderate interest in phone, email and Internet based services, while interest in social media based services was lower. In these two groups, sociodemographic variables were not predictive of interest in telemedicine [26].

On the other hand, the pediatric context is particularly favorable for telemedicine services: parents and patients are young and familiar with new technologies. This is confirmed by our findings: most participants had a high level technological profile, both in terms of availability of technological tools and in terms of used functions, including videocalls, messaging services and social networks. Such profile, together with the elevated cultural level of our population, represents a fertile environment for the development and the spreading of telemedicine services.

Regarding the interest towards telemedicine of the surveyed population, our findings are in line with the results of most published surveys, reporting a positive attitude and a strong interest towards telemedicine in the pediatric context. Great satisfaction was expressed towards a telemedicine service for child and adolescent mental health [27]. In a randomized controlled trial, families of obese children showed a high level of satisfaction towards a telemedicine group intervention [28]. A survey conducted on patients’ families regarding the teleconsultation service provided at the Royal Children’s Hospital in Melbourne showed a high level of satisfaction, most families declaring that they had received the same standard of care as in a face-to-face consultation [29]. Families are interested in telemedicine-based follow up of their children after a visit at the emergency department [30]. A positive perception towards telemedicine, with a variable degree of preference for face-to-face consultation was detected among families of children undergoing a genetic consultation [31] and a screening for retinopathy of prematures [32].

In our study population, three quarters of patients were affected by a chronic disease, and had frequent contacts both with the hospital and with their family pediatrician. At the multivariate analysis, presence of a chronic illness and disease severity were not associated with willingness to use telemedicine services. Despite this lack of association, as a matter of fact, pediatric patients with chronic diseases, often requiring frequent contacts with health care institutions, may benefit from televisits and telemonitoring, with a direct impact on costs, in particular those associated with hospital readmission [33]. Enrolled families accounted cost saving as an interesting advantage of the implementation of telemedicine services.

Preferred telemedicine functions concerned communication with the hospital, in terms of logistics (data transmission, appointment scheduling) and clinical communication (distance communication in case of emergency).

Such functions may have an interesting impact on quality of life, which can be positively affected by telemedicine services [33] through reducing the need of in-person visits (time saving was the most regarded item among the advantages of telemedicine in our population), speeding up processes, empowering patients and strengthening their feeling of security.

On the other hand, those who were less keen on the use of telemedicine mainly did not trust the technologies which would be involved in the provision of telemedicine services. Despite the overall high digital literacy of the target population, issuing an app for health or a service of televisits warrants a strong program of education, directed towards patients, patients’ families and health care professionals, in order to guarantee adequate use and implementation.

Interestingly, owning a tablet was associated with both study outcomes, showing that tablets may have a great potential for the delivery of telemedicine services.

Due to their size allowing a mix of portability and good image quality, tablets represent an ideal media for distance communication and for the use of apps dedicated to health care. Tablets have been widely used for telemedical purposes, targeting both health care professionals [34–37] and patients [38–40].

Our study has several strengths: first, the study population is large, thus increasing precision of estimates. Moreover, we included clinical information in the survey, which allowed us to study the potential association of telemedicine attitude and expectations with variables such as severity of the disease and chronicity, which have been rarely taken into account in such studies.

Our study also has a number of limitations. First, we did not acquire information on people declining to answer to the questionnaire, thus the responding population might be selected towards those more interested in telemedicine. Secondarily, we investigated education level and employment as proxies for the socio-economic status of the enrolled families. To this regard, even though the estimation of the socio-economic status through these variable may not be precise, our population might have been selected towards those with a high level of education and employment, thus leading to an overestimation of technological profiles and of the attitude towards telemedicine. However, confounding by socio-economic variables was taken into account in the multivariable analysis. Thirdly, our results are likely influenced by the specific context (patients attending a tertiary care pediatric hospital in central Italy), and their generalizability to other settings may be limited.

## Conclusion

Families of pediatric patients with different clinical problems are keen to embark in telemedicine programs, irrespective of severity of disease or chronicity, and of distance from the hospital. Technological profiles of these families are largely sufficient to meet basic requirements for telemedicine services based on videocalls and dedicated apps. Since the market offers many commercial solutions with different levels of complexity for telemedicine programs, the only determinant for implementation regards feasibility, including presence of appropriate infrastructures in telemedicine hospital providers, and availability of personnel for telemedicine services.

## Additional file

Additional file 1: Questionnaire on the attitude towards telemedicine among families of pediatric patients. (DOCX 25 kb)

## Abbreviations

ASA: American Society of Anesthesiologists
CI: Confidence interval
MesH: Medical subject heading
OR: Odds ratio
PC: Personal computer
SD: Standard deviation
VAS: Visual analog scale
