# Supplementary material for: What drives attitude towards telemedicine among families of pediatric patients? A survey
Source: BMC Pediatr. 2017 Jan 17;17:21. doi: 10.1186/s12887-016-0756-x (PMC5240275; doi:10.1186/s12887-016-0756-x)
Supplement: Additional file 1: — Questionnaire on the attitude towards telemedicine among families of pediatric patients. (DOCX 25 kb) [file 12887_2016_756_MOESM1_ESM.docx]

Questionnaire on the attitude towards telemedicine

Questionnaire N. ________

among families of pediatric patients

*Parents’ data*

Sex: M F Age, years:________ Nationality: Italian  Other

Education: Primary  Secondary  High School  University

Employment: employed  unemployed

Postal code:

*Child’s data*

Sex: M F

Age, years:_____; months:_____

Number of hospital admissions during last year (including Day Hospital and outpatients visits):

0  <5  >5

Number of visits by family pediatrician during last year: 0  <5  >5

*Use of Internet*

Which of the following devices do you own (more than one answer allowed)?

smartphone  PC  tablet  smart TV  none

Do you own a blog? YES  NO

On which social network do you have a profile/account (more than one answer allowed)?

Facebook  Twitter  LinkedIn  Instagram  Google+  None

Do you use any of the following messaging softwares (more than one answer allowed)?

Whatsapp  Skype  Facebook messenger  other  none

Do you use any software for videocalls (like Skype or Facetime)? YES  NO

You connect to the Internet through (more than one answer allowed):

Computer  Smartphone  Tablet  other  I don’t use the Internet

Have you ever used an app (application) for smartphone or tablet? YES  NO

Have you ever used an app for smartphone or tablet dedicated to health? YES  NO

Have you ever searched for health information on the Internet? YES  NO

*Telemedicine*

*Telemedicine allows to receive clinical health care from a distance, even from home, and to have a video connection with a doctor through the use of computers of smartphones.*

**If available, would you use the following telemedicine services:**

a televisit service (i.e. a visit with a doctor

in videoconference) YES  NO

an app providing telemedicine services YES  NO

**Which level of usefulness would you assign**

**to the following services:**

None A bit Sufficient Moderate Much

An app allowing communication

with other parents of children

A diary for recording the child's health status

An app for scheduling medical visits

A service for televisits (i.e. a visit with a doctor

in videoconference)

A service for distance monitoring (telemonitoring)

of clinical parameters through specific devices

A service for transmitting

telemonitoring data to the doctor

A service for consulting a doctor

in case of emergency

Reminders for medical visits

A service providing transmission

of health data from the hospital

Reminders for therapy

A newsletter on health promotion

**Which level of importance would you assign**

**to the following potential advantages of**

**telemedicine:**

None A bit Sufficient Moderate Much

Time saving

Cost saving

Empowerment of patients

Empowerment of families

**Which level of fear on telemedicine do you have**

**regarding the following issues:**

None A bit Sufficient Moderate Much

lack of trust regarding the use of monitoring

devices without the presence of a physician

privacy issues

difficulty in using technological devices     
